# Utilization of immunological ratios in HIV: Implications for monitoring and therapeutic strategies

**DOI:** 10.1097/MD.0000000000037354

**Published:** 2024-03-01

**Authors:** Emmanuel Ifeanyi Obeagu, Getrude Uzoma Obeagu

**Affiliations:** a Department of Medical Laboratory Science, Kampala International University, Kampala, Uganda; b School of Nursing Science, Kampala International University, Kampala, Uganda.

**Keywords:** CD4/CD8 ratio, HIV, inflammation ratios, MLR, monitoring, NLR, therapeutic strategies

## Abstract

Human immunodeficiency virus (HIV) infection remains a significant global health concern, necessitating ongoing research and innovation in the quest for improved disease management. Traditional markers for monitoring HIV progression and the effectiveness of antiretroviral therapy have limitations in capturing the intricate immune responses and inflammatory dynamics in people with HIV. In recent years, the concept of inflammation ratios has gained prominence as a valuable tool for assessing and understanding the complex interplay between inflammation, immune function, and HIV. In this abstract, we provide an overview of the emerging field of utilizing inflammation ratios in the context of HIV and its implications for disease monitoring and therapeutic strategies. These ratios, such as the CD4/CD8 ratio, neutrophil-to-lymphocyte ratio, and monocyte-to-lymphocyte ratio, offer a more comprehensive assessment of an individual’s immune status and inflammatory state. By exploring the clinical implications of inflammation ratios, including their potential to predict disease complications and guide personalized treatment approaches, this publication sheds light on the potential benefits of incorporating inflammation ratios into routine HIV care. Furthermore, we emphasize the importance of ongoing research in this field to further refine our understanding of the utility and significance of inflammation ratios in improving the lives of people with HIV.

## 1. Introduction

Human immunodeficiency virus (HIV) infection continues to be a major global health challenge, with millions of individuals affected by the virus and new infections occurring worldwide.^[1–4]^ Over the decades, significant progress has been made in the management of HIV, particularly with the advent of highly effective antiretroviral therapy (ART).^[5]^ However, the complexity of HIV infection and its impact on the immune system demand innovative approaches for disease monitoring and therapeutic strategies.^[6]^ In recent years, the concept of utilizing inflammation ratios has emerged as a promising avenue for assessing and addressing the intricate interplay between inflammation, immune dysfunction, and HIV progression.

Inflammation ratios represent a novel and dynamic tool that enables healthcare providers, researchers, and individuals living with HIV to gain deeper insights into the underlying inflammatory processes associated with the infection. These ratios involve the measurement of specific biomarkers and their relative proportions, offering a more comprehensive and nuanced understanding of the immune status and inflammatory state of people with HIV (PWH).^[7]^

This paper delves into the utilization of inflammation ratios in the context of HIV and explores their implications for monitoring disease progression and designing tailored therapeutic strategies. We will examine the most commonly used inflammation ratios, their clinical significance, and their potential to reshape the way we manage HIV. By shedding light on this emerging field, we aim to contribute to the ongoing efforts to enhance the care and quality of PWH.

## 2. Methodology

A lot of research search databases were utilized in writing the paper such as PubMed/MEDLINE, Scopus, Web of Science, Embase, Google Scholar, Researchgate and Cochrane Library using a list of keywords and phrases related to these concepts such as HIV, immunological markers, CD4/CD8 ratio, immune response, monitoring HIV treatment, therapeutic interventions.

## 3. Inflammation ratios in HIV

Despite the substantial progress made in the treatment of HIV with the advent of ART, the complex interplay between the virus, the immune system, and inflammation continues to pose challenges for both clinicians and researchers. Monitoring disease progression and determining the effectiveness of treatment in HIV necessitates a more nuanced understanding of the immune and inflammatory status of PWH. In this context, inflammation ratios have emerged as a promising and innovative approach.^[8]^

Inflammation ratios, as the name suggests, involve the comparison of specific biomarkers associated with inflammation and immune function. They provide a more comprehensive view of the immune and inflammatory landscape of PWH. This paper explores the concept of inflammation ratios in the context of HIV, discussing their clinical relevance, potential implications for monitoring disease progression, and their role in the development of tailored therapeutic strategies.^[9]^

## 4. Commonly utilized inflammation ratios

Several inflammation ratios have gained recognition in the field of HIV research, each offering a unique insight into the immune and inflammatory status of affected individuals:

1. *CD4/CD8 ratio*: This ratio is calculated by dividing the number of CD4^+^ T cells (helper T cells) by the number of CD8^+^ T cells (cytotoxic T cells). A low CD4/CD8 ratio is indicative of immune dysfunction and has been associated with advanced disease progression and increased inflammation.^[10]^
2. *Neutrophil-to-lymphocyte ratio (NLR*): NLR measures the balance between neutrophils, which are pro-inflammatory cells, and lymphocytes, which play a key role in immune defense. Elevated NLR is associated with systemic inflammation and has been linked to adverse outcomes in HIV.^[11,12]^
3. *Monocyte-to-lymphocyte ratio (MLR*): MLR involves comparing monocyte levels, which are involved in immune activation and inflammation, with lymphocyte levels. Elevated MLR is associated with a higher risk of opportunistic infections and disease progression.^[13]^

## 5. CD4/CD8 ratio in HIV

The CD4/CD8 ratio is a crucial immunological parameter used in the context of HIV infection and AIDS (acquired immunodeficiency syndrome). This ratio represents the balance between 2 types of T-lymphocytes, or T cells, in the immune system: CD4^+^ T cells (also known as helper T cells) and CD8^+^ T cells (cytotoxic T cells).^[14]^

In individuals with HIV, monitoring the CD4/CD8 ratio is essential for several reasons:

1. *Assessing immune health*: CD4^+^ T cells are a critical component of the immune system and play a central role in coordinating the immune response. They are the primary target of HIV, which infects and depletes these cells. CD8^+^ T cells, on the other hand, are involved in immune responses against infected cells. The CD4/CD8 ratio provides insight into the relative health and functionality of the immune system.^[15–17]^
2. *Monitoring disease progression*: In PWH, the CD4/CD8 ratio tends to decrease over time as the virus progressively damages CD4^+^ T cells. A declining ratio is indicative of more advanced disease and a weakened immune system. By tracking changes in this ratio, healthcare providers can monitor disease progression and determine when to initiate or adjust ART.^[18]^
3. *Predicting complications*: A low CD4/CD8 ratio is associated with an increased risk of various HIV-related complications, including opportunistic infections and non-AIDS-related conditions such as cardiovascular disease, neurocognitive impairments, and certain cancers. Monitoring the ratio can help identify individuals at higher risk for these complications.^[17]^
4. *Treatment decision-making*: The CD4/CD8 ratio is considered in conjunction with CD4 cell counts and viral load when making treatment decisions for PWH. While ART is the cornerstone of HIV management, the CD4/CD8 ratio can provide additional information to guide treatment initiation, evaluate treatment efficacy, and inform decisions about when to start or modify therapy.^[17]^
5. *Immunological recovery*: As individuals with HIV initiate ART and experience immune recovery, the CD4/CD8 ratio often improves. This improvement is a positive indicator of treatment effectiveness and the restoration of immune function.^[19]^

The CD4/CD8 ratio is a valuable biomarker in the management of HIV. It provides insights into the immune status, disease progression, and the overall health of individuals living with HIV. Monitoring this ratio, in conjunction with other laboratory and clinical parameters, is essential for making informed decisions about treatment and care for PWH.^[17]^

## 6. NLR in HIV

The NLR is a hematologic parameter used to assess the balance between 2 different types of white blood cells in the bloodstream: neutrophils and lymphocytes.^[20]^ This ratio has gained attention in various medical contexts, including in the management of HIV infection.^[21]^ NLR can serve as a valuable marker for evaluating the systemic inflammatory state and immune response in PWH. Here’s how NLR is relevant in the context of HIV:

### *Inflammation and immune activation*:

HIV infection is characterized by chronic immune activation and systemic inflammation. Elevated levels of inflammation can contribute to disease progression and the development of HIV-related complications. NLR is a simple and cost-effective way to assess inflammation, as neutrophils are often associated with acute inflammation, while lymphocytes play a critical role in adaptive immune responses.^[22,23]^

### *Predictive value*:

Elevated NLR is often associated with adverse outcomes in various diseases, including HIV. In the context of HIV, a high NLR has been linked to more advanced disease, a greater risk of opportunistic infections, and non-AIDS-related conditions, such as cardiovascular disease and certain malignancies. Monitoring NLR can help identify individuals at higher risk for these complications.^[24,25]^

### *Treatment response*:

NLR can also provide insights into the effectiveness of ART. Effective ART typically leads to a reduction in inflammation and immune activation. Therefore, a decreasing NLR over time may be indicative of a positive response to treatment and improved immune function.^[26]^

### *Comprehensive assessment*:

NLR is often used in conjunction with other laboratory and clinical parameters, such as CD4 cell counts and viral load, to assess the overall health and immune status of PWH. A comprehensive evaluation that includes NLR can guide treatment decisions and disease management.^[27]^

### *Research and prognostic tool*:

NLR is being studied as a potential prognostic tool in HIV research. It can help researchers and clinicians better understand the relationship between inflammation, immune function, and disease outcomes in PWH.^[28,29]^

The NLR is a valuable biomarker for assessing inflammation and immune activation in PWH. Monitoring NLR alongside other clinical and laboratory parameters can aid in evaluating disease progression, predicting complications, and assessing the response to ART. This information contributes to a more comprehensive understanding of HIV and informs treatment decisions and care strategies.^[30]^

## 7. MLR in HIV

The MLR is another hematologic parameter used to assess the balance between 2 different types of white blood cells in the bloodstream: monocytes and lymphocytes.^[31]^ In the context of HIV infection, MLR can provide valuable insights into the immune status and inflammation levels in individuals living with the virus. Here’s how MLR is relevant in the context of HIV:

1. *Immune activation and inflammation*: HIV infection is characterized by chronic immune activation and systemic inflammation. Monocytes play a crucial role in immune activation, as they are involved in the inflammatory response. Elevated levels of monocytes and, consequently, a high MLR can be indicative of increased inflammation in individuals with HIV.^[22,32]^
2. *Predictive value*: High MLR has been associated with more advanced HIV disease and an increased risk of opportunistic infections. Monitoring MLR can help identify individuals who may be at a higher risk of developing complications related to HIV infection.^[33]^
3. *Treatment response*: MLR can also be used as a marker to assess the effectiveness of ART. Effective ART typically leads to a reduction in inflammation and immune activation, which may be reflected in a decreased MLR. Monitoring changes in MLR over time can provide insights into the response to treatment.^[34]^
4. *Comprehensive assessment*: MLR is often considered alongside other clinical and laboratory parameters, such as CD4 cell counts, viral load, and the NLR, to assess the overall health and immune status of PWH. A comprehensive evaluation, including MLR, can guide treatment decisions and disease management.^[35]^
5. *Research and prognostic tool*: Researchers are studying the potential of MLR as a prognostic tool in HIV. It can help in understanding the complex interplay between immune activation, inflammation, and disease progression in PWH.^[36]^

The MLR is a valuable biomarker for assessing inflammation and immune activation in PWH. Monitoring MLR in conjunction with other clinical and laboratory parameters contributes to a more comprehensive understanding of the immune status and overall health of PWH. This information is valuable for making informed treatment decisions and managing the complexities of HIV infection.^[37]^

## 8. Clinical implications of inflammation ratios in HIV

Inflammation ratios, such as the CD4/CD8 ratio, NLR, and MLR, have important clinical implications in the management of HIV infection.^[38]^ These ratios offer valuable insights into the immune status and inflammatory state of PWH, and their clinical significance extends to several aspects of HIV care:

1. *Disease monitoring*: Inflammation ratios serve as additional markers for monitoring HIV disease progression and the effectiveness of ART. A declining CD4/CD8 ratio is indicative of advanced disease and may prompt clinicians to adjust treatment regimens. Increasing NLR and MLR can signal ongoing immune activation and inflammation.^[38]^
2. *Predicting complications*: Elevated inflammation ratios, such as NLR and MLR, are associated with a higher risk of HIV-related complications, including opportunistic infections, cardiovascular disease, neurocognitive impairments, and non-AIDS-related malignancies. Monitoring these ratios can help identify individuals at higher risk for such complications and facilitate early intervention.^[39]^
3. *Personalized treatment*: The use of inflammation ratios can guide personalized treatment strategies. For example, individuals with high inflammation ratios may benefit from adjunctive anti-inflammatory interventions alongside standard ART. Treatment decisions can be tailored based on these ratios to optimize therapeutic outcomes.^[39]^
4. *Immunological recovery*: Improvements in inflammation ratios, such as a rising CD4/CD8 ratio, can signify immune recovery in response to effective ART. Monitoring these changes over time can confirm the success of treatment in restoring immune function.^[40]^
5. *Comprehensive assessment*: Integrating inflammation ratios into routine clinical assessments provides a more holistic view of an individual’s immune and inflammatory status. Clinicians can use these ratios in combination with traditional markers like CD4 cell counts and viral load to make more informed decisions about treatment and care.^[41]^
6. *Research and prognostic tool*: Ongoing research on inflammation ratios in HIV informs our understanding of their clinical significance and potential as prognostic tools. Research may lead to the development of risk assessment models based on these ratios, which could aid in patient management and early intervention.

The clinical implications of inflammation ratios in HIV are significant. These ratios offer a multifaceted approach to assessing the immune and inflammatory status of individuals with HIV, and they provide valuable information for clinicians in terms of monitoring disease progression, predicting complications, tailoring treatment, and evaluating the effectiveness of therapy. Continued research in this field will likely refine our understanding of how best to utilize inflammation ratios to improve the overall care and outcomes of PWH.^[42]^

## 9. Seminal studies on CD4/CD8 ratios

The CD4/CD8 ratio is a measure of the balance between 2 subtypes of T lymphocytes, which are important components of the immune system. CD4 T cells are often referred to as helper T cells, while CD8 T cells are cytotoxic T cells. The CD4/CD8 ratio is used as an indicator of immune function and has been studied in various contexts, including HIV/AIDS, autoimmune diseases, and cancer.^[19,43,44]^

### 9.1. CD4 and CD8 T lymphocyte interplay in controlling T-cell homeostasis (1996)

This study, published in the journal Immunological Reviews, delves into the role of CD4 and CD8 T cells in maintaining immune system balance and homeostasis.^[45]^

### 9.2. CD4/CD8 ratio and immune activation as independent predictors of HIV disease progression (1999)

This study, published in the Journal of Acquired Immune Deficiency Syndromes, focuses on the prognostic value of the CD4/CD8 ratio in HIV/AIDS patients and how it relates to disease progression.^[46]^

### 9.3. Changes in CD4 and CD8 T cell subsets in response to HIV infection (2000)

Published in the Immunology Letters, this study investigates the alterations in CD4 and CD8 T cell subsets during HIV infection, shedding light on the immunological changes associated with the disease.^[47]^

### 9.4. CD4^+^/CD8^+^ ratio and CD8^+^ counts predict CD4^+^ counts in Ugandans with HIV (2003)

This study, published in the Journal of Acquired Immune Deficiency Syndromes, explores the relationship between CD4/CD8 ratios and CD4 counts in PWH in Uganda.^[48]^

### 9.5. CD4/CD8 ratio and risk of Kaposi sarcoma in homosexual men with HIV (2007)

Published in the Journal of the National Cancer Institute, this study investigates the association between the CD4/CD8 ratio and the risk of Kaposi sarcoma in PWH.^[49]^

It’s important to note that research in this field is ongoing, and newer studies may have been published since my last update. It’s advisable to search for more recent literature to stay current on advancements in our understanding of CD4/CD8 ratios and their implications in various health conditions.^[49]^

## 10. Immunological non-responders

Immunological non-responders (INRs) refer to a subset of individuals living with HIV/AIDS who, despite receiving ART, show suboptimal or limited immune reconstitution.^[50]^ In other words, their CD4 T-cell counts remain low despite effective viral suppression. The immune response, as measured by CD4 T-cell count, is typically expected to improve in individuals on ART, but INRs exhibit a limited or no increase in CD4 counts.^[50]^ The criteria for defining immunological non-response can vary, but it often involves individuals who fail to achieve a certain threshold of CD4 T-cell recovery despite having undetectable viral loads on ART. Immunological non-response is associated with an increased risk of AIDS-related and non-AIDS-related complications, as the compromised immune function may leave individuals more susceptible to opportunistic infections and other health issues.^[51]^ The reasons behind immunological non-response are multifactorial and not fully understood. Factors contributing to this phenomenon may include late initiation of ART, preexisting damage to the immune system, ongoing immune activation, co-infections, and host genetics. Research is ongoing to better understand the mechanisms underlying immunological non-response and to develop strategies to improve immune recovery in these individuals. Some studies have explored interventions such as immunomodulatory therapies or treatment intensification strategies. Managing INRs can be challenging.^[52]^ Clinicians may need to closely monitor these individuals, consider additional diagnostic tests to identify potential underlying issues, and may explore different treatment approaches. As our understanding of the heterogeneity of HIV infection increases, there is a growing interest in personalized medicine approaches to tailor treatment strategies based on individual patient characteristics. It’s important to note that advancements in HIV research occur regularly, and new findings may have emerged since my last update in January 2022. For the most current information, it’s recommended to consult recent scientific literature or speak with healthcare professionals specializing in HIV care.

## 11. Nuances of several monocyte subsets in HIV

In the context of HIV infection, monocytes, which are a type of white blood cell, play a crucial role in the immune response.^[53]^ Monocytes can be classified into different subsets based on the expression of surface markers. Understanding the nuances of these monocyte subsets in the context of HIV is important for comprehending the complex interactions between the virus and the immune system. Here are some key points about several monocyte subsets in HIV.

### 11.1. Classical monocytes (CD14^++^CD16^−^)

These are the most abundant monocytes in the peripheral blood. In HIV infection, the number of classical monocytes may be altered, and their functionality can be affected. Classical monocytes are involved in phagocytosis and are essential for initiating the inflammatory response.^[54]^

### 11.2. Intermediate monocytes (CD14^+^CD16^+^)

Intermediate monocytes have a phenotype that falls between classical and non-classical monocytes. Their numbers can increase in chronic inflammatory conditions, including HIV infection. Intermediate monocytes are known to produce pro-inflammatory cytokines.^[55]^

### 11.3. Non-classical monocytes (CD14^low^CD16^+^)

Non-classical monocytes are involved in tissue surveillance and repair. In HIV infection, the number of non-classical monocytes may increase, and they are thought to contribute to chronic immune activation and inflammation. Non-classical monocytes are associated with the dissemination of HIV to tissues.^[56]^

## 12. Functional changes

HIV infection can lead to functional changes in monocytes, including altered cytokine production and impaired phagocytic capacity. Persistent immune activation in HIV may contribute to the dysregulation of monocyte subsets and their functions.^[57,58]^

## 13. Role in HIV persistence

Monocytes and their subsets may play a role in HIV persistence, as they can serve as reservoirs for the virus. Infected monocytes can traffic the virus to various tissues, contributing to viral persistence and potentially reactivating the infection.^[59]^

## 14. Therapeutic implications

Understanding the dynamics of monocyte subsets in HIV infection is relevant for therapeutic strategies. Some studies explore interventions aimed at modulating monocyte activation or trafficking to reduce inflammation and improve overall immune function.^[60]^

Research in this area is ongoing, and the nuances of monocyte subsets in HIV are an active area of investigation. Advances in our understanding of the immune response to HIV may contribute to the development of targeted therapies to modulate monocyte function and mitigate the chronic immune activation associated with HIV infection. For the latest information, it’s advisable to refer to recent scientific literature.

## 15. Research and future directions of inflammation ratios in HIV

Research into inflammation ratios in the context of HIV continues to evolve and holds promise for improving our understanding of HIV pathogenesis, disease progression, and potential therapeutic interventions. Future research in this field is likely to focus on several key areas:^[6]^

1. *Refinement of biomarker panels*: Researchers may explore the development of more comprehensive biomarker panels that include a combination of inflammation ratios (e.g., CD4/CD8 ratio, NLR, MLR) and other relevant markers. These panels could provide a more nuanced assessment of immune and inflammatory status in HIV, enabling a more accurate prediction of disease outcomes.^[6]^
2. *Early disease detection*: Investigating the potential of inflammation ratios as early disease markers is essential. Identifying individuals at higher risk of rapid disease progression based on these ratios could lead to earlier interventions and more effective disease management.^[6]^
3. *Impact on treatment strategies*: Research may continue to explore the influence of inflammation ratios on treatment strategies. This includes understanding how to tailor treatment regimens based on an individual’s specific inflammation profile and evaluating the benefits of anti-inflammatory therapies as adjuncts to standard ART.^[6]^
4. *Longitudinal studies*: Long-term studies that track inflammation ratios over extended periods will help elucidate their utility as prognostic tools. These studies can provide insights into how these ratios change over time and whether they correlate with clinical outcomes.^[6]^
5. *Comparative analyses*: Comparative analyses of inflammation ratios in different HIV subtypes, populations, and geographical regions can reveal variations in disease dynamics and the role of host genetics, which may lead to more targeted interventions.^[6]^
6. *Impact on comorbidities*: Research may explore the relationship between inflammation ratios and the development of non-AIDS-related comorbidities, such as cardiovascular disease, neurocognitive disorders, and cancers. Understanding how inflammation contributes to these conditions is crucial for comprehensive HIV care.^[6]^
7. *Biomarker-based risk assessment*: Future research could focus on developing risk assessment models that incorporate inflammation ratios and other clinical markers. These models could help clinicians identify individuals at higher risk for complications and tailor interventions accordingly.^[6]^
8. *Immunotherapies and vaccines*: Investigating the impact of inflammation ratios on the efficacy of immunotherapies and HIV vaccine candidates is an emerging area. Understanding how these ratios influence immune responses to therapeutic interventions is crucial for advancing treatment options.^[6]^
9. *Precision medicine*: The concept of precision medicine in HIV care may involve considering inflammation ratios as part of an individual’s treatment plan. Research may explore how to personalize treatment strategies based on an individual’s unique inflammation profile.^[6]^
10. *Interplay with microbiome*: The relationship between inflammation ratios and the gut microbiome, which plays a significant role in HIV-related inflammation, is an area of growing interest. Research in this field could lead to a more holistic understanding of HIV pathogenesis.^[6]^

Research into inflammation ratios in HIV is a dynamic field with significant potential for improving our understanding of HIV disease progression and patient management. Future research directions are likely to focus on refining these ratios as diagnostic and prognostic tools, exploring their impact on treatment strategies, and advancing our knowledge of HIV-related complications and comorbidities. These endeavors have the potential to enhance the quality of care and the long-term outcomes for individuals living with HIV.^[61–63]^

## 16. Conclusion

Inflammation ratios provide a novel perspective on the immune status and inflammatory state of PWH. As we strive for more comprehensive and personalized HIV care, these ratios offer valuable insights that can help guide treatment decisions and improve the overall health outcomes of PWH. Ongoing research in this area will continue to shape the future of HIV management and care.

## Author contributions

**Conceptualization:** Emmanuel Ifeanyi Obeagu.

**Methodology:** Emmanuel Ifeanyi Obeagu.

**Supervision:** Emmanuel Ifeanyi Obeagu.

**Visualization:** Emmanuel Ifeanyi Obeagu.

**Writing – original draft:** Emmanuel Ifeanyi Obeagu, Getrude Uzoma Obeagu.

**Writing – review & editing:** Emmanuel Ifeanyi Obeagu, Getrude Uzoma Obeagu.
